# How pharmacology can aid in the diagnosis of mental disorders

**DOI:** 10.1007/s00210-024-03413-z

**Published:** 2024-09-04

**Authors:** Roland Seifert, Bastian Schirmer, Johanna Seifert

**Affiliations:** 1 Institute of Pharmacology, Hannover Medical School, Carl-Neuberg-Str. 1, 30625 Hannover, Germany; 2 Department of Psychiatry, Social Psychiatry and Psychotherapy, Hannover Medical School, Carl-Neuberg-Str. 1, 30625 Hannover, Germany

**Keywords:** Depression, Schizophrenia, Bipolar disorder, NE/5-HT enhancers, mGPCR antagonists, Calcium channel blockers, Sodium channel blockers, Lithium

## Abstract

The precise diagnosis of mental disorders constitutes a formidable problem. Mental disorders are currently diagnosed based on clinical symptoms, which are often subjective. Various drug classes, traditionally referred to as “antidepressants,” “antipsychotics” and “mood stabilizers” are then used empirically to treat affected patients. The previous decade has witnessed an increasing extension of the use of drug classes beyond their traditional indications (e.g., “antidepressants” in the treatment of anxiety disorders). Therefore, we would like to initiate a discussion in the pharmacological and psychiatric research communities on an alternative classification of mental disorders: Instead of using the traditional categorical classification of mental disorders physicians should rather diagnose symptoms (e.g., anhedonia) without bias to a traditional categorization (e.g., depression). The appropriate most effective drugs are then selected based on these symptoms. Depending on the responsiveness of the patient towards a given drug X, the disease should be classified, e.g., as drug X-responsive disease. This approach will also help us elucidate the still poorly understood molecular mechanisms underlying mental disorders, i.e., drugs can also be viewed and used as molecular diagnostic tools. In several fields of medicine, drugs are already used as molecular diagnostic tools. Thus, there is already precedence for the concept proposed here for mental disorders.

## Objective diagnostic tools are available for many diseases

Some diseases are easy to diagnose. This is especially true for diseases with easily objectifiable diagnostic criteria. For example, we can simply measure arterial blood pressure; if blood pressure exceeds a certain value, arterial hypertension is diagnosed. Renal failure is diagnosed by determining creatinine clearance, while a cytological, histological, and molecular analysis of tumor cells may lead to the diagnosis of a malignant disease. To determine the cause of bacterial sepsis, pathogens circulating in the blood stream are cultivated, and stroke is diagnosed after a neurological exam and brain imaging. And a fracture is easily diagnosed by an X-ray.

## Fluid diagnoses in psychiatry — a result of missing objective diagnostic tools

The diagnosis of mental disorders, however, is much more complicated. Until now, most of psychiatric diagnostic techniques rely on the exploration of the patients in terms of their behavior, mood, and intellectual abilities. Of course, imaging techniques, neurological exams, biochemical, and toxicological analyses support the diagnostic procedures, but in most cases, with exception of dementia, there are no unequivocal biomarkers of the underlying mental disorders (Insel et al. 2010; Wakefield 2014; Lozupone et al. 2019). Instead, we end up with an array of (largely subjective) clinical symptoms that are bundled together into diagnostic entities classified in the International Classification of Disease (ICD-10) or the Diagnostic and Statistical Manual of Mental Disorders (DSM-5) (Becker 2021; Evans et al. 2021; Scull 2021). Major psychiatric disease entities that largely rely on solely clinical criteria are depression, bipolar disorder, schizophrenia, anxiety disorders, obsessive–compulsive disorders, autism, and personality disorders. These diseases are then treated empirically with various psychiatric drug classes, traditionally referred to as “antidepressants,” “antipsychotics,” and “mood stabilizers.” Psychiatry is beginning to respond to these problems. One example is the call for eliminating the term “schizophrenia” as a diagnostic entity, because of the heterogenous presentation of this disease (Zick et al. 2022). In South Korea, the term “schizophrenia” has now been replaced by the more neutral and less stigmatizing term “attunement disorder” (Won Cho et al. 2018).

## Pharmacology responds to the erosion of diagnosis and therapy of psychiatric diseases by a mechanistic drug class nomenclature

While the current classification system of diagnoses fulfills the requirement for a systematic structure in psychiatry and the requirements of health insurances, hospitals, out-patient clinics, and psychiatrist to assign any given patient a specific “diagnostic box,” the implementation of this classification system is not always as clear cut as one might hope. First, it can be observed that many psychiatric diagnoses are seemingly arbitrary and fluctuate with each visit of the patient, also depending on the respective physician performing the evaluation. This instability of psychiatric diagnoses may especially be seen in patients suffering from psychosis. A patient diagnosed with a psychosis, due to mania (bipolar disorder) during one acute episode, may be diagnosed with schizophrenia in the next and perhaps schizoaffective disorder in the following episode (Wood et al. 2021). A common denominator in the treatment of all of disorders presenting with symptoms of psychosis is that they generally respond to treatment with antipsychotic drugs. However, psychotic symptoms also respond to treatment with drugs other than antipsychotic drugs depending on the underlying mental disorder. In so, psychotic symptoms in major depression also respond to treatment with antidepressants (Oliva et al. 2024) and psychotic symptoms in bipolar mania respond to treatment with lithium (Crapanzano et al. 2022). Therefore, as will be outlined below, we have recently proposed to abandon traditional names of drug classes such as “antipsychotics” and “antidepressants” (Seifert and Schirmer 2020a). In fact, in the instructions for authors of this journal, the use of mechanistically oriented drug class terms is now mandatory (Seifert and Schirmer 2021).

## Symptoms in many psychiatric diseases overlap, and so does the clinical use of drugs

There is also substantial evidence that the genetic influences underlying psychiatric disorders are subject to significant overlap, as is the case for the at first glance seemingly unrelated mental disorders schizophrenia and autism (St Pourcain et al. 2018). Additionally, patients often present with symptoms of various diseases, e.g., depression, anxiety disorder, plus obsessive–compulsive disorder and are diagnosed with multiple mental disorders (Forman-Hoffman et al. 2018; Plana-Ripoll et al. 2019; Buitenen et al. 2020). In fact, most patients diagnosed with depression suffer from at least one comorbid mental disorder, most commonly neurotic, stress-related, and somatoform disorder (i.e., ICD-10: F4-diagnoses) (Steffen et al. 2020). It is often difficult to attribute a certain psychopathological feature to a specific disease. For example, a patient suffering from obsessive–compulsive disorder exhibiting poor insight may be mistakenly diagnosed with a psychotic disorder due to the delusional presentation of symptoms. On the other hand, patients formerly diagnosed with obsessive–compulsive disorder appear to have a higher risk of later developing schizophrenia, raising the question if the obsessions and compulsions were in fact symptoms of their prodromal stage (Kim et al. 2023).

## Empirically, the use of drugs in psychiatry for diseases beyond the original indication has broadened over time

The sedimented dogma that depression is treated with antidepressants, bipolar disorder is treated with mood stabilizers, and schizophrenia is treated with antipsychotics has become outdated and is progressively thwarted by the notion that multiple disorders share a common psychopathological basis (Insel et al. 2010; Caspi and Moffitt 2018). One starting point for the erosion of these dogmata was the observation that certain patients with depression do not sufficiently respond to conventional antidepressants but benefit from augmentation with antipsychotics (Wright and Lippmann 1987; Zhou et al. 2015; Yan et al. 2022). The alkali ion lithium not only exerts superior effects as mood stabilizer in bipolar disorder, but also as augmentation drug in depression. Evidence regarding lithium’s use as adjunct therapy in schizophrenia is less robust (Leucht et al. 2015; Siskind et al. 2018; Strawbridge et al. 2019; Nuñez et al. 2022). To render issues more complicated, several “antiepileptics” such as lamotrigine, valproic acid, phenytoin, and pregabalin can also be effective in certain patients suffering from the afore-mentioned three clinical entities (Amann et al. 2007; Melvin et al. 2008; Mehndiratta and Sajatovic 2013; Zheng et al. 2017). Again, depending on the underlying disorder and individual symptom presentation, the effects of antiepileptic drugs are pronounced. Valproate, for example, has proven effective in the treatment of excitement and aggression in patients with schizophrenia. However, these effects are based only on open (vs. placebo-controlled) random-controlled trials (Wang et al. 2016).

It has been empirically observed that antidepressants, antipsychotics, mood stabilizers, and antiepileptics could exert beneficial effects in numerous other psychiatric disorders including anxiety disorders, obsessive–compulsive disorders, and autism spectrum disorders (Hefner et al. 2022). Perhaps even more remarkably, drugs not primarily deemed useful in the treatment of neuropsychiatric disorders are being considered for these indications. For example, recent studies indicate that the classic antibacterial drug minocycline could be used as adjunctive drug in schizophrenia and depression, constituting a fundamental breach with our conventional classification of drug classes and diseases alike (Panizzutti et al. 2023). These observations, together with advances in our understanding of mental disorders, call into question the current categorical psychiatric diagnoses and provide indications for mental disorders to be considered as very individual mixtures (continua) of symptoms (Markon et al. 2011; Tomczyk et al. 2023).

## The result of these developments in pharmacology and psychiatry is confusion: where should we go?

These longstanding developments have led us to a perplexing situation with respect to clinical diagnosis of psychiatric diseases, proper designation of drug classes, and proper clinical use of these drug classes. The mismatch between designation of drug classes and their clinical use has reached an extent where patient communication and specific literature searches in databases have become difficult (Ghaemi 2015; Seifert and Schirmer 2020b). Table 1 provides some examples of drugs with an evident mismatch between indication and traditional drug class designation as compared to current clinical use.

**Table 1:** Examples of drugs that are currently used in indications beyond the traditional use

Drug / drug class	Traditional indication	Traditional drug class designation	Alternative indication	Mechanistic drug class designation	Reference
Selective serotonin reuptake inhibitors (SSRI)	Depression	Antidepressant	Management of behavioral symptoms in frontotemporal lobar degeneration	SSRI *or* NE/5-HT enhancers	(Nisar et al. 2021)
Pregabalin	Epilepsy, neuropathic pain	Antiepileptic	Generalized anxiety disorder	CCB	(Baldwin et al. 2013)
Risperidone	Schizophrenia	Antipsychotic	Obsessive–compulsive disorder	mGPCR antagonists	(Hollander et al. 2003)
Lithium	Bipolar disorder, mania	Mood stabilizer	Pathological gambling	Alkali ions	(Chaim et al. 2014)
Lisdexamfetamine	Attention deficit hyperactivity disorder (ADHD)	Psychostimulant	Binge eating disorder	NE/DA enhancers	(Guerdjikova et al. 2016)
Minocycline	Bacterial infections	Antibacterial drug	Adjunctive for schizophrenia and depression	Tetracyclines (inhibitors of bacterial protein synthesis)	(Panizutti et al. 2023)

The use of a drug beyond its traditional indication is also referred to as drug repurposing and is a powerful approach to take advantage of the knowledge on existing drugs. Sometimes, serendipity leads to successful repurposing, sometimes “trial and error” and sometimes mechanism-based research. In any case, with the advancement of drug repurposing, traditional drug class designations become incorrect and cause a lot of irritation among scientists, doctors, and patients alike. By using mechanistically driven drug class designations, such irritation can be avoided

To solve this unsatisfying situation, numerous attempts have been made to diagnose psychiatric diseases in the same manner as cardiovascular, kidney, tumor, or infectious diseases, that is with the aid of defined objective parameters, such as changes in brain biochemistry, changes in the structure of the brain, or biomarkers in the blood (Bellivier et al. 2013; Lozupone et al. 2019; Kennis et al. 2020). Despite very intense efforts, however, no valid and clinically useful diagnostic system for mental disorders, apart from dementia, has emerged thus far.

## Are drugs for the treatment of psychiatric diseases as ineffective as often claimed? Perhaps, we approached the problem the wrong way, and drugs are more effective than we thought

Another nagging problem related to the overall situation is the unsatisfying outcome of many clinical studies assessing the effects of drugs on psychiatric diseases. In many studies, no or only small beneficial effects of drugs for important diseases such as depression, bipolar disorder, and schizophrenia were noted (Papakostas et al. 2016; Kamenov et al. 2017; Huhn et al. 2019). It is well possible that, for these clinical studies, simply the wrong patients had been selected, i.e., those who did not respond to the examined drug. Had a different patient group been selected, perhaps drug efficacy would have been greater. Participation in clinical trials requires informed consent, and the ability to provide consent decreases with the severity of the underlying mental disorder (Helmchen 1981). So, while antidepressant drugs appear more efficacious in patients with more severe depression (Kirsch et al. 2008), this group of patients may be largely excluded from clinical trials. Due to these strict inclusion criteria, patients who participate in clinical drug trials may significantly differ from “real-life” collectives, which appears to be a particular issue in psychiatry (Tan et al. 2022). Because of these highly controlled clinical trials, the overall value of psychopharmacological treatments has been questioned. Furthermore, the development of innovative psychotropic drugs by the pharmaceutical industry was slowed down due to low profit potential and, probably, due to overfocussing on sedimented psychopathological beliefs (Ghaemi 2022; Miller and Raison 2023; Cosgrove et al. 2023). Moreover, the field of psychiatry appears to have to lowest success rate in clinical drug development, rendering it particularly unattractive to drug companies (Zhu 2021).

## Off-label use of drugs without scientific basis is not a solution to the therapeutic dilemma

Another consequence of this unsatisfying situation is that drugs for mental disorders need to be tested empirically in numerous patients to reveal if they respond to a certain pharmacotherapy or not. It is quite common that even within a given drug class, several individual drugs must be tested individually to find the best possible treatment. Sometimes, patients respond to a combination of drugs with different mechanisms of action. Quite often, the therapeutic regimes found to be most effective differ substantially from schemes using traditional drug class designations (e.g., antidepressants, antipsychotics, antiepileptics, mood stabilizers). In so, psychotropic drugs are often used in “off-label” indications (Vijay et al. 2018; Hefner et al. 2022).

## A mechanistic nomenclature of drugs for psychiatric diseases, an important step towards rational thinking and untangling the conundrum

Increasing dissociations between traditional drug class designations and the actual clinical use of these drugs in pharmacology were comprehensively discussed in previous reviews (Seifert 2018; Seifert and Schirmer 2020b). It was proposed that the traditional classification of drugs, focusing on clinical indications, should be discontinued. Instead, a mechanistic nomenclature should be adopted, allowing unbiased assignment of drug classes to emerging clinical uses. With respect to psychopharmacology, it was proposed to abandon the term “antidepressants” and replace it with the more neutral term “norepinephrine/serotonin (NE/5-HT) enhancers.” The term “antipsychotics” should be replaced by the more appropriate term “antagonists at multiple G-protein-coupled receptors” (mGPCR antagonists). Moreover, it was suggested that the terms “mood stabilizers” and “antiepileptics” should be deleted entirely. Instead, drug classes with mood-stabilizing and/or antiepileptic properties should be named according to their mechanism of action, e.g., sodium channel blockers (SCB) or calcium channel blockers (CCB). In the case of lithium (with still largely unknown mechanism of action), the term “alkali ions” as a drug class was proposed. This term alludes to lithium distributing in the organism like sodium, thus causing adverse effects in many organ systems because of its ubiquitous distribution. This new mechanistic nomenclature has already been introduced into an English and a German pharmacology textbook (Seifert 2019, 2021). In these textbooks, it was already noted that the therapeutic effects of a given class of psychiatric drugs in clinically heterogenous conditions point to a common underlying pathophysiological mechanism. How this mechanistic nomenclature may translate into everyday practice, especially when communicating with patients, is a current matter of debate.

## How pharmacology can help to solve the diagnostic and therapeutic problems in psychiatry: the perspective of the psychiatrist and individual patient

**Fig. 1:**
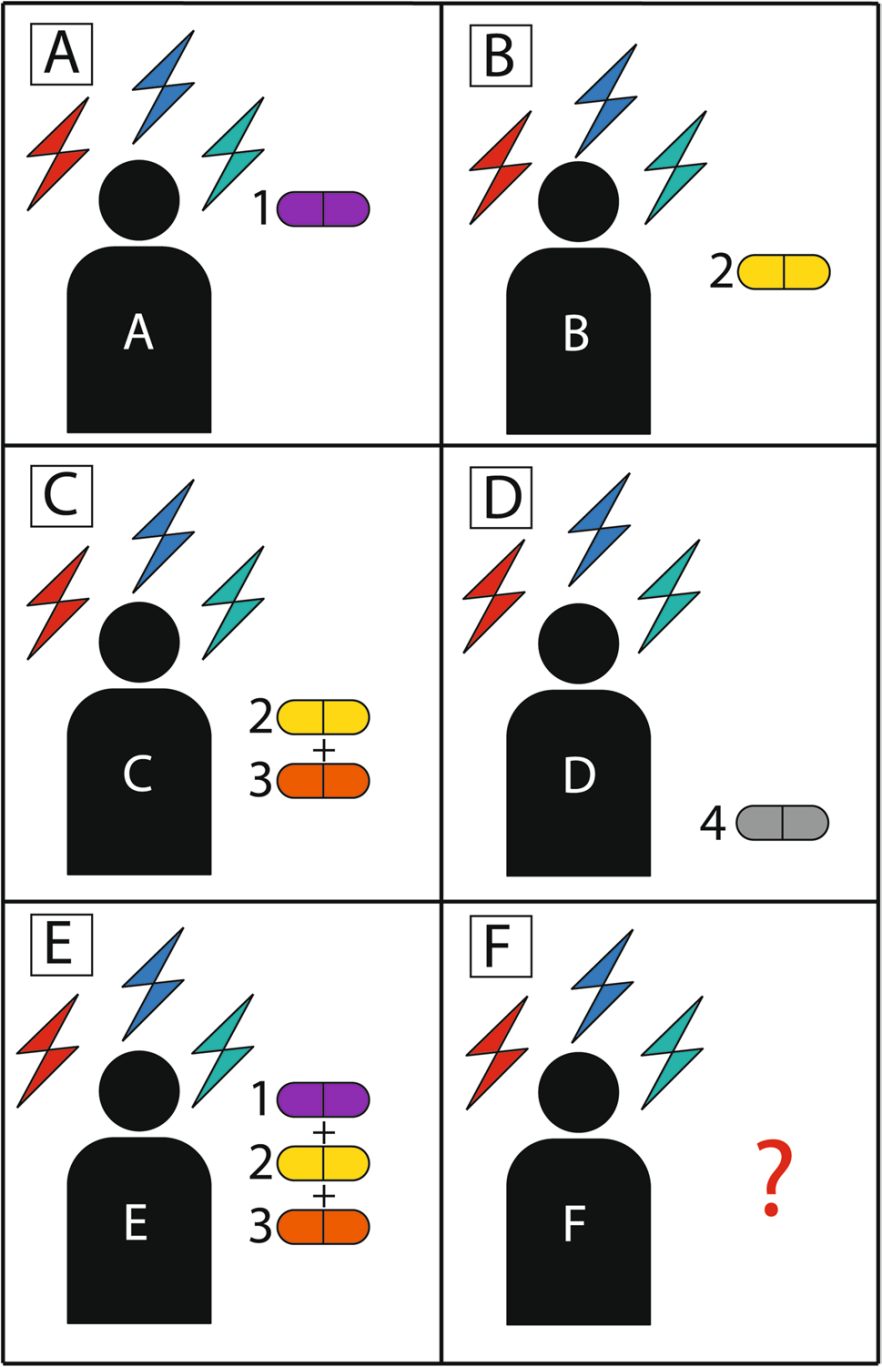
Patients with the same symptoms but different pharmacological diagnoses. The figure illustrates six different patients (**A**–**F**) who all have the same symptoms as symbolized by the different-colored flashes of lightning. Patients in **A**, **B**, and **D** all respond to different drugs, and patients in **C** and **E** respond to combinations of drugs with different mechanism of action. The patient in **F** is not responsive to drugs at all. Thus, based on pharmacological responsiveness of the symptoms, six different pharmacological diagnoses can be made, despite presentation with similar clinical symptoms. The different drug responsiveness of the symptoms indicates that all six patients may have a different underlying pathophysiology. Thus, drugs can be used to dissect psychiatric diseases mechanistically. Here, six different diseases are illustrated, referred to as drug 1-responsive disease, drug 2-responsive disease, drug 2 + 3-responsive disease, drug 4-responsive disease, drug 1 + 2 + 3-responsive disease, and drug 1–4-non-responsive disease. The advantage of this classification is that it facilitates testing of specific drugs in patients who have not yet responded sufficiently to a given drug or combination of drugs

Based on the issues discussed above, it is evident that some of the current diagnostic and therapeutic problems in psychiatry are related to terminology and how we approach an individual patient. Therefore, we propose the following strategy to address the problems:

In the beginning, we should make a descriptive list of psychopathological symptoms, designated as different-colored arrows in Fig. 1. The severity of symptoms, their duration, and impact on a patient’s quality of life must also be taken into consideration. To do so, clinicians may use standardized psychometric tests such as the Becks-Depression-Inventory (BDI) and the Positive and Negative Syndrome Scale (PANSS). In the second step, based on the prevalent symptoms, we select (guess) the best drug (drug 1) for this patient (Fig. 1A). However, it is possible that another patient with the same symptoms does not respond optimally to drug 1, so he or she will end up with drug 2 (Fig. 1B). Perhaps, in yet another patient showing the same symptoms, drug 2 shows some effect, but we must augment this effect with another drug (drug 3, Fig. 1C). In the case of inefficiency, we may either test another drug (drug 4, Fig. 1D) or combine drugs 1, 2, and 3 (Fig. 1E). Drugs 1–4 each have different molecular mechanisms of action. There are, of course, multiple other possible permutations of these considerations, the worst (but unfortunately not uncommon) permutation being drug resistance of a given combination of symptoms (Fig. 1F). Of course, other aspects such as the occurrence of adverse drug reactions, drug-drug interactions, general drug tolerability, and pharmacokinetics must also be considered, which may limit the use of certain drugs.

Thus, although the symptoms of different patients look very similar to the clinician’s eye, they exhibit vast differences in individual responsiveness to various drugs. Such heterogeneity in drug responses towards a given combination of clinical symptoms is also an explanation for the poor outcome of many clinical studies in the field of psychiatry. Conversely, this scenario also implies that the pathophysiological basis underlying a given combination of clinical symptoms is different. In other words, we can also use drugs for psychiatric diseases to probe the underlying pathophysiology. Once we understand the pathophysiology of a given psychiatric disease better, it will also be easier to develop effective drugs for this disorder.

## A given drug can be effective in different diseases, pointing to a common pathophysiology: network medicine

**Fig. 2:**
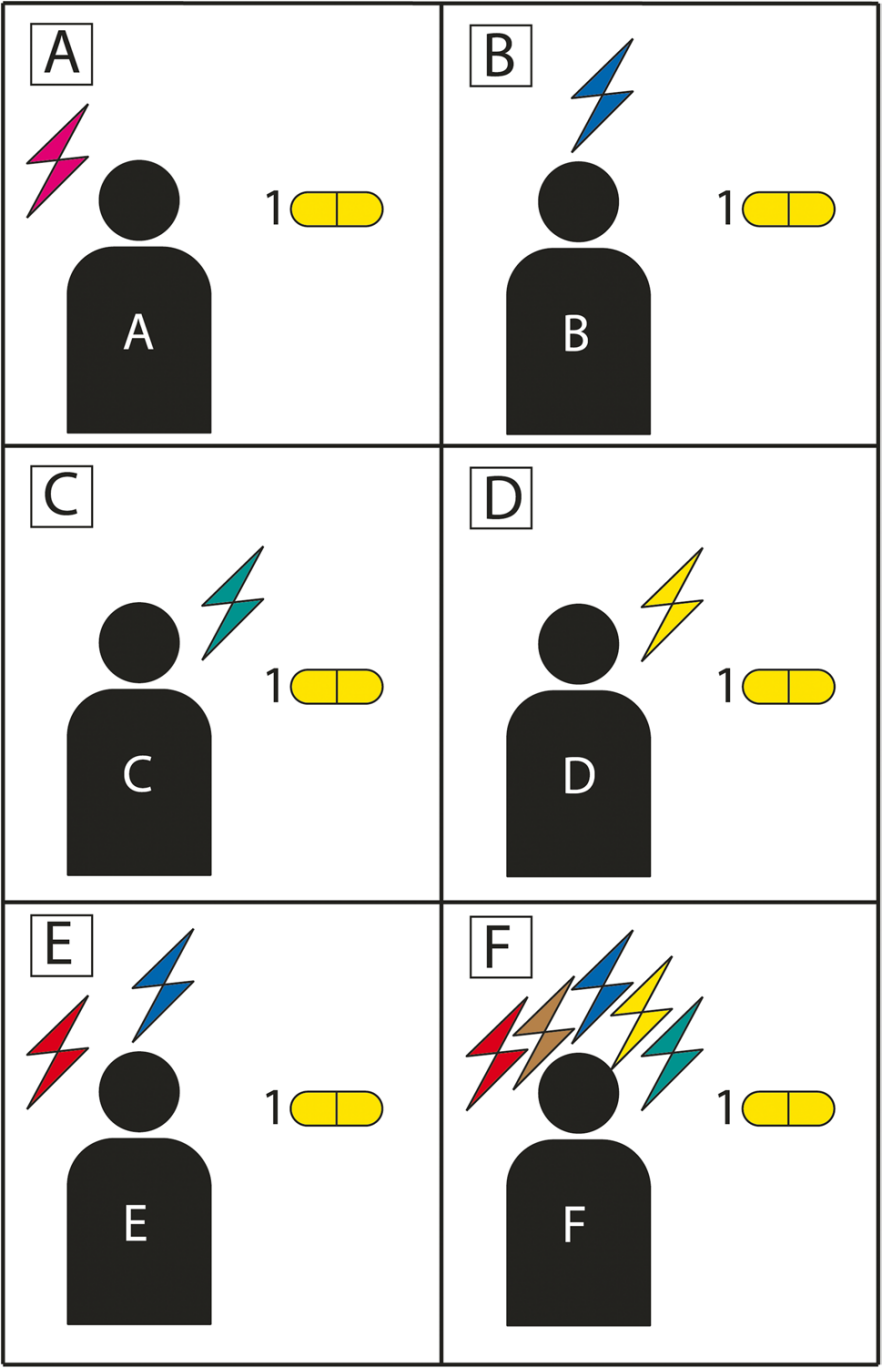
Multiple patients with different symptoms but the same pharmacological diagnosis. The figure illustrates six different patients (**A**–**F**) who all have a different type or number of symptoms, as symbolized by different-colored flashes of lightning. The symptoms of patient E (**E**) correspond to the sum of the symptoms of patient A (**A**) and patient B (**B**). Patient F (**F**) has symptoms that overlap with the symptoms of all patients **A**–**E**. Thus, although all patients depicted here have a unique pattern of clinical symptoms, i.e., in traditional terms six different clinical diagnoses, pharmacologically they have the same diagnosis. The identical drug responsiveness of different clinical symptoms indicates that all six patients have a common underlying pathophysiology. Thus, drugs can be used to dissect psychiatric diseases mechanistically. The disease illustrated here should be referred to as drug 1-responsive disease. The advantage of this classification is that it mechanistically links diseases together that clinically would be considered completely different entities

The importance of drugs as diagnostic tools for psychiatric diseases is also highlighted when we consider the opposite scenario, i.e., when a given drug is effective in clinically diverse disorders. This scenario is depicted in Fig. 2. Here, patients A to D exhibit different main clinical symptoms, symbolized again by different-colored flashes of lightning (Fig. 2A–D). Patient E shows the combination of the symptom of patients A and D, and patient F exhibits overlapping symptoms of patients 1–5 plus additional symptoms. Although all the patients differ from each other clinically, nonetheless, they all respond to the same drug (“drug 1”). This scenario also helps to explain why clinical studies in the field of psychiatric diseases are so frustrating, because usually patients with similar symptoms are grouped together. The scheme shown in Fig. 2. also shows that clinically very heterogeneous diseases may have a common pathophysiological basis, and this is unmasked by a given drug. This approach to disease classification is also implemented within the concept of network medicine. In this concept, diseases are classified according to signaling pathways they have in common (Silverman et al. 2020; Maiorino and Loscalzo 2023). This concept encompasses many disease groups including psychiatric diseases (Silbersweig and Loscalzo 2017).

## So far, the literature on pharmacological treatment of psychiatric diseases was ordered based on diagnoses rather than molecular mechanisms of drugs

This approach has also important implications for how we should perform literature searches in the future. Traditionally, searches focus on a given disease and the treatment options. In the future, literature searches could be useful to focus on the search for clinical symptoms and their responsiveness to a given drug. For example, antidepressants may not provide equal relief in all symptom domains of depression (e.g., depressed mood, anhedonia, feelings of guilt). However, when examining the individual symptoms of depression, selective serotonin reuptake inhibitors (SSRIs) consistently alleviate depressed mood, while other symptoms of depression are not beneficially impacted by SSRI use (Hieronymus et al. 2016).

The expected efficacy of a certain drug in alleviating a certain symptom must of course also be considered when exploring a patient’s psychopathology and hierarchizing symptoms. Further consideration must be given to the severity of symptoms. The perhaps most well-known discussion of this nature is the significantly lower responsiveness of non-severe depression to antidepressants in comparison to severe depression (Khan et al. 2005). Such finding suggests that pharmacological approaches do not represent the ideal treatment strategy for all patients. The consideration of specific symptoms and their responsiveness to psychotropic drugs has also relevance for the future design of clinical studies. Evidently, retrospective analysis of the scientific literature to match a given symptom with a drug is not trivial because traditionally, research papers and clinical papers alike were written based on diagnoses according to ICD or DSM, which could have caused a bias.

## A new design of pharmacological studies in psychiatry: fundamental changes in our way of thinking are required

We realize that the models shown in Figs. 1 and 2 may be deemed heretical by some psychiatrists and scientists alike, and it is certainly not our intention to propagate an excessive off-label use of psychotropic drugs, but our suggestion may provide a strategy to improve the current diagnostic and therapeutic dilemma of psychiatry. One could design sufficiently large clinical trials in which patients with a set of similar symptoms are treated with different drug regimes in parallel as shown in Fig. 1 or sequentially. Once responders to a given drug have been identified, one could analyze specifically those biochemical parameters that are known to be targeted by a given drug. This procedure, integrating drugs both as therapeutic and diagnostic tools, could refine the classification and therapy of psychiatric diseases.

To address the scenario shown in Fig. 2, it would be necessary to identify those patients with diverse clinical conditions who respond well to a given drug. In these patients, one could then analyze biochemical parameters targeted by the specific drug. Even drug-driven metabolomic techniques could be developed to better understand the pathophysiology of clinically heterogenous psychiatric diseases.

**Fig. 3:**
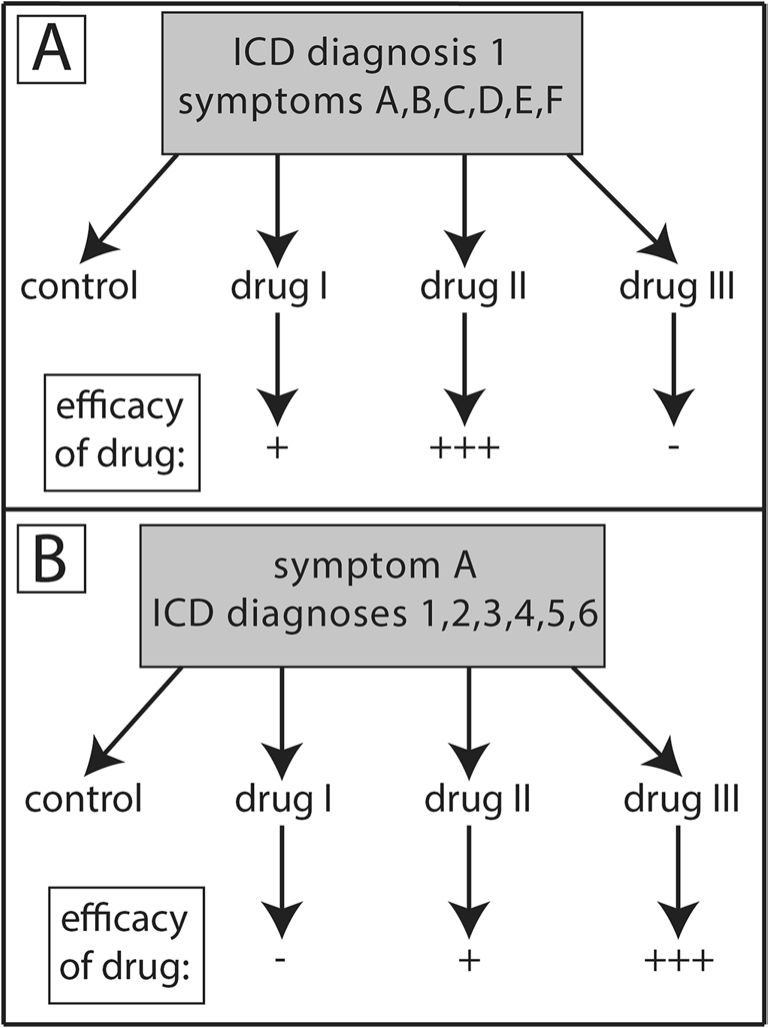
Current and future design of pharmacological studies in the field of psychiatry. **A** Traditionally, drugs are tested pharmacologically in disease entities based on the ICD system. Patients with a given ICD diagnosis usually have different clinical symptoms. In classic studies, various drugs are compared with each other regarding their efficacy in each disease. Based on the results of many such studies, recommendations regarding specific drugs for a given ICD diagnosis are made. **B** In future clinical studies, patients should not be grouped according to a specific ICD diagnosis but rather according to a predominant clinical symptom. This symptom may be part of several ICD diagnoses. In the next step, the efficacy of various drugs on a given symptom is assessed. It is very likely that such studies, which have rarely performed so far, will yield a completely different drug efficacy result than the traditional clinical study scheme

The patient-oriented description of drug efficacy in psychiatric diseases shown in Figs. 1 and 2 has consequences for the future design of pharmacological studies in this field (Fig. 3). Traditionally, pharmacological studies are ICD- or DSM-diagnosis-driven, i.e., patients with a given ICD/DSM diagnosis are used as starting population for a clinical study (Fig. 3A). In such a traditional study design, various drugs are compared with each other, and differences in efficacy are observed. Usually, the most effective drug is then recommended for the treatment of large populations of patients in therapeutic guidelines issued by learned societies. However, the problem with this approach is that there are many poor responders or non-responders.

Therefore, an alternative approach for future clinical studies is proposed (Fig. 3B). Patients with a dominant clinical symptom should be grouped together as a starting population for a clinical study. Non-conventionally and counterintuitively for traditional thinking, these patients may be labeled with very different ICD diagnoses (conventional diagnostic boxes). In these patients, the efficacy of various drugs will be assessed largely the same way as in the traditional manner (Fig. 3A), but as readout, not the overall improvement of a specific disease will be assessed but rather the improvement of a given clinical symptom. It is predicted that this approach will yield a very different drug efficacy scheme than the traditional approach. Most likely, the difference in efficacy among the different drugs in the symptom-driven approach will be much more pronounced than in the ICD-driven approach. It is hoped that in this way, it will be easier to identify suitable drugs for patients suffering from a dominant psychiatric symptom. At the same time, if we identify highly effective drugs, this will tell us that a specific symptom is mediated by a specific neuronal signaling pathway modulated by a given drug. And if know the mechanism of this drug, we can learn much about the pathophysiology of a given symptom.

The pharmaceutical industry will benefit from a redesign of clinical studies as well. Most likely, drugs that were previously designated as ineffective in a traditional ICD indication and never reached the market will show efficacy in a new study design. Such successes will increase the number of drugs reaching the market, increasing the number of drugs available to psychiatrists. In return, successful new drugs on the market will foster basic and clinical research on psychiatric drugs both in academia and industry.

## From traditional pharmacology to reverse pharmacology: drugs as tools to decipher pathophysiology and generate new diagnostic entities in psychiatry

**Fig. 4:**
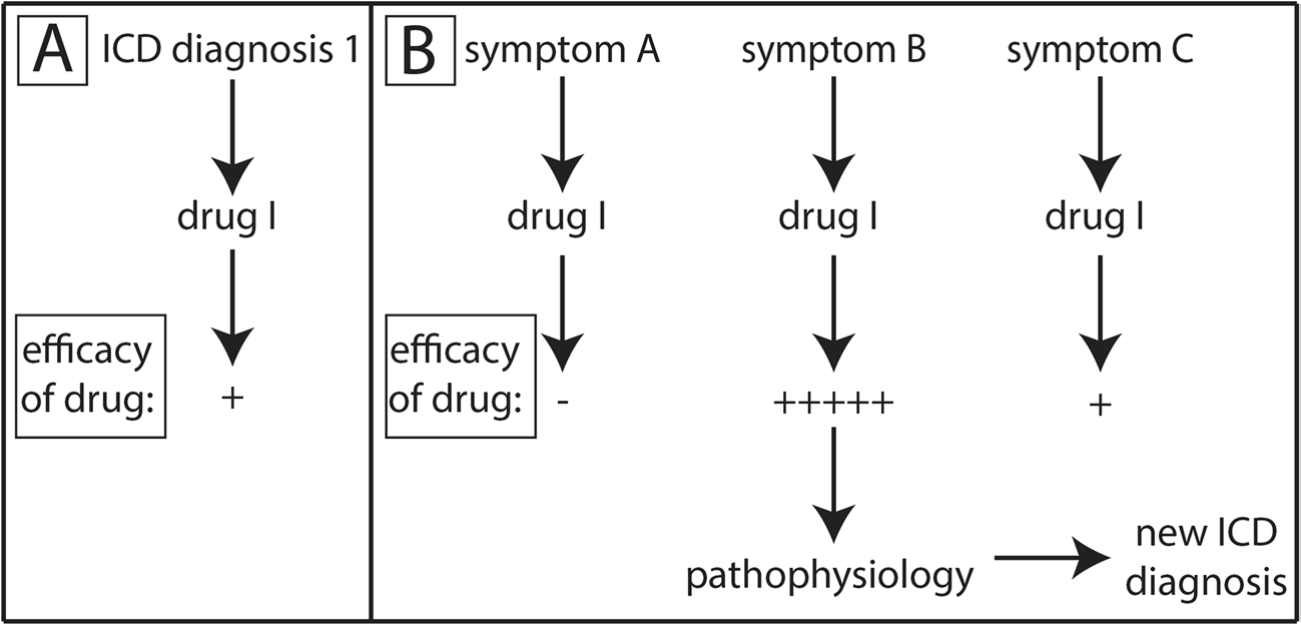
Traditional versus reverse pharmacology. **A** In traditional pharmacology, we start out with a disease, based on a known and established ICD diagnosis. This is a “box” deeply rooted in all layers of health systems globally. ICD diagnoses are particularly important for reimbursing hospitals and practitioners by health insurances. So, without ICD diagnosis, there is no payment. These constraints shape the thinking of all professional participants in health care, education, and science. Based on the ICD diagnosis, an appropriate drug will be selected. Particularly for psychiatric diseases, the efficacy of drugs is often only moderate, resulting in a broadly held claim about general inefficacy of psychopharmacology. This assumption is not necessarily true but a result of our inappropriate thinking and methodological approach. **B** In reverse pharmacology, we start out with specific symptoms (without ICD diagnosis and actually encompassing several ICD diagnoses, see Fig. 3) that differentially respond to a given drug. Of particular interest in reverse pharmacology are symptoms that respond very well to a given drug. We can deduce the underlying pathophysiology of the symptom if we know the molecular mechanism of action of the drug. Thus, a drug, in addition to being used as therapeutic agent, will be used as diagnostic tool. Based on the pathophysiology, we can then generate a new ICD diagnosis such as “drug I-responsive symptom B.” Most of the literature has been assembled according to traditional pharmacology. Thus, it will be very difficult to use previous literature to develop the concept of reverse pharmacology

Figure 4 shows traditional pharmacology versus reverse pharmacology. In traditional pharmacology, we start out with a specific disease with a specific ICD (Fig. 4A). Based on the diagnosis, we treat the disease with a given drug, too often only with very moderate efficacy. In the reverse pharmacology approach, we start out with various symptoms that do not necessarily fall under the same ICD category (Fig. 4B). We will probably observe that a particular clinical symptom is responding very well to this drug, whereas other symptoms are less responsive. If we know the molecular mechanism of action of the specific drug, we can then deduce a specific pathophysiological mechanism underlying this symptom. If the case is compelling, in the last step, we can then generate a new ICD diagnosis. Thus, in reverse pharmacology, drugs are not only used to effectively treat symptoms but also as diagnostic tools to generate completely new diagnostic entities (boxes).

## Using drugs as diagnostic tools is not uncommon in medicine: some examples

There is precedence for using drugs as diagnostic tools in various fields of medicine. For example, the muscarinic receptor agonist pilocarpine is used to diagnose cystic fibrosis (De Boeck et al. 2017). For diagnosis of myasthenia, the short-acting acetylcholine esterase inhibitor edrophonium is used (Pasnoor et al. 2018). Indomethacin, a cyclooxygenase inhibitor, is used to diagnose specific forms of headache (Villar-Martínez et al. 2021). Oxygen is used both as diagnostic tool and therapy for cluster headache (Burish 2024). Lastly, in oncology, protein kinase inhibitors with efficacy for specific target proteins are increasingly used to classify malignant diseases at the molecular level (Zhou et al. 2024), allowing for personalized therapy. Thus, given the paucity of objective diagnostic tools in psychiatry, drugs with a defined molecular mechanism of action are most welcome additions to the diagnostic toolkit. Moreover, by analogy to malignant diseases, personalized drug therapy in psychiatry, empirically applied for many patients, would become a more rational mechanistic basis.

## What needs to be done in the future: revision of ICD and the ATC classification of drugs and educating the next generation of researchers and clinicians alike

A major road block towards implementation of the suggestions made herein is the way how psychiatrists and pharmacologists are currently trained, i.e., an abandonment of deeply rooted diagnostic and therapeutic entities and an unbiased de-novo re-analysis of psychiatric diagnoses and pharmacological treatments is required. Perhaps, in the end, an improved classification and treatment of psychiatric diseases will emerge from these efforts. A simple way to implement this diagnostic strategy is to add a “specifier” as is already provided for several diagnostic entities in the ICD-11 that provides additional information regarding drug responsiveness (e.g., recurrent depressive disorder, severe with psychotic symptoms, responsive to venlafaxine, lithium, and olanzapine). In so, the currently employed diagnostic classification system would not be completely abandoned, but the specification of drug responsiveness would represent an additional piece of information, which may also aid treatment in the future. It should also be noted that several efforts have already been put forth to implement more dimensional (as opposed to categorical) approaches in the diagnosis of mental disorders. The ICD-11 now uses a dimensional approach to classify personality disorders using five different domains (i.e., negative affectivity, detachment, dissociality, disinhibition, anankastia) to describe the prominent personality traits (ICD-11, WHO 2019/2021). While in personality disorders, these traits are in general not considered to be responsive to drug treatment, such an approach could provide useful in other mental health disorders. Specifiers indicating the patient’s symptoms could further be complemented with responsiveness to different (non)pharmacological treatment (e.g., schizophrenia, first episode with primarily catatonic features, responsive to lorazepam and electroconvulsive therapy). However, although this process may be painful at the beginning, no action is not an alternative. The detrimental effects of the diagnostic dilemma in psychiatry and the mismatch of drug class designation and the actual clinical use signal the urgent need for change. Other groups have recognized this dilemma as well and proposed a neuroscience-based classification of psychoactive drugs (Zohar et al. 2014, 2015; Zohar and Kasper 2016; Zohar and Levy 2022) or a “subway map” of psychoactive drugs (Zhou et al. 2022).

The proposed change in nomenclature of drugs for the treatment of psychiatric diseases is currently being discussed at the level of the IUPHAR (International Union of Pharmacologists) and the World Health Organization (School of INN; https://extranet.who.int/soinn/). Evidently, this proposal must be discussed in the various psychiatric societies. At the level of medical education in Germany, the Institute for Medical and Pharmaceutical Exam Questions (IMPP) has already implemented the proposed nomenclature change in the medical curriculum as a basis for future changes in diagnostics and therapy of psychiatric diseases (“Gegenstandskataloge,” https://www.impp.de/pruefungen/ allgemein/gegenstandskataloge.html). Representatives of the DGPPN (*Deutsche Gesellschaft für Psychiatrie und Psychotherapie, Psychosomatik und Nervenheilkunde* — German Association for Psychiatry, Psychotherapy and Psychosomatics) and the DGPT (*Deutsche Gesellschaft für Experimentelle und Klinische Pharmakologie und Toxikologie —* German Society for Experimental and Clinical Pharmacology and Toxicology) were part of the peer review group. Noteworthy, another medical discipline facing a paradigm shift towards a more mechanistical disease classification is oncology with cancer more and more being viewed as a disease of the genome instead of an organ (André et al. 2024).

It will also be necessary to adjust the Anatomical Chemical Therapeutic (ATC) classification of drugs that, traditionally, has a strong focus on therapeutic indications rather than mechanisms of action (https://www.who.int/tools/atc-ddd-toolkit/atc-classification). The current change in indication of psychiatric drugs renders it difficult to locate specific drugs properly in pharmacoeconomic publications such as the German Drug Prescription Report (“Arzneiverordnungs-Report 2022”, Ludwig et al. 2023). For example, pregabalin, widely used for the treatment of anxiety disorders, is listed therein under “Epilepsies” (chapter 24). Along the same line, quetiapine is listed under “Drugs with antipsychotic effects” (chapter 22), although it is also used for the treatment of depression.

## Conclusions: time for change how we think about pharmacology and psychiatry

We know that change in medicine takes a lot of time. We have recently epitomized the sluggishness of the pharmacological and medical community to respond to necessary changes in textbooks and the scientific literature using the poison hydrogen cyanide and the antihypertensive and antipsychotic drug reserpine as paradigms (Ludwig and Seifert 2024; Misera and Seifert 2024). But not responding now to apparent challenges would deprive us from the opportunity to build the intellectual framework for a sustainable and comprehensive pharmacology and psychiatry.

Proper classification of mental disorders is a matter of current debate. The increasing mismatch between clinical use of psychotropic drugs and their traditional designation has resulted in an initiative to rename psychiatric drug classes mechanistically. This allows an unbiased use of a given drug to treat psychopathological features of psychiatric conditions. Abandoning traditional clinical symptom-based classification of psychiatric diseases and implementation of a drug responsiveness-based classification may, in the long run, improve effectiveness of drug treatment of psychiatric diseases and shed new light on the underlying pathophysiology of these diseases. In the next round of revision of the ICD regarding psychiatric diseases, pharmacologists should be integrated to implement some of the suggestions made in this paper.

## Data Availability

No datasets were generated or analysed during the current study.
